# Development of a novel cell-based, In-Cell Western/ERK assay system for the high-throughput screening of agonists acting on the delta-opioid receptor

**DOI:** 10.3389/fphar.2022.933356

**Published:** 2022-09-26

**Authors:** Junaid Asghar, Liaque Latif, Stephen P. H. Alexander, David A. Kendall

**Affiliations:** ^1^ Faculty of Pharmacy, Gomal University, Dera Ismail Khan, Pakistan; ^2^ School of Life Sciences, Faculty of Medicine and Health Sciences, Medical School, QMC, University of Nottingham, Nottingham, United Kingdom

**Keywords:** delta-opioid receptor, ERK, fluorescence, In-Cell Western, high-throughput screening, GPCR drug discovery

## Abstract

**Background:** Extracellular signal-regulated kinases (ERKs) are important signaling mediators in mammalian cells and, as a result, one of the major areas of research focus. The detection and quantification of ERK phosphorylation as an index of activation is normally conducted using immunoblotting, which does not allow high-throughput drug screening. Plate-based immunocytochemical assays provide a cheaper and relatively high-throughput alternative method for quantifying ERK phosphorylation. Here, we present optimization steps aimed to increase assay sensitivity and reduce variance and cost using the LI-COR In-Cell Western (I-CW) system in a recombinant CHO-K1 cell line, over-expressing the human delta-opioid receptor (hDOPr) as a model.

**Methods:** Cells cultured in 96-well microassay plates were stimulated with three standard/selective DOPr agonists (SNC80, ADL5859, and DADLE) and a novel selective DOPr agonist (PN6047) to elicit a phospho-ERK response as an index of activation. A number of experimental conditions were investigated during the assay development.

**Key results:** Preliminary experiments revealed a clearly visible edge-effect which significantly increased assay variance across the plate and which was reduced by pre-incubation for 30 min at room temperature. ERK phosphorylation was detectable as early as 1 min after agonist addition, with a distinct peak at 3–5 min. Optimization of the cell seeding densities showed that 25,000 cells per well have the lowest basal phospho-ERK response and an optimal agonist ERK1/2 signal. Pre-incubation with apyrase (an ATPase) did not reduce the basal or agonist responses. All agonists produced concentration-dependent increases in phospho-ERK activation, and pertussis toxin was able to attenuate these ERK responses. Naltrindole, which is a selective DOPr antagonist, was able to antagonize the DOPr-mediated ERK activation of the ligands.

**Conclusion:** We have developed an optimization protocol and highlighted a number of considerations when performing this high-throughput fluorescence immunocytochemical (ICC) assay measuring ERK phosphorylation in the human DOPr. The optimized protocol was found to be a more conducive option for the screening of delta agonists. This provides a basis for additional assay development to investigate opioid pharmacology. This protocol should be widely applicable for measuring ERK phosphorylation in any cell line and investigating other protein targets in GPCR drug discovery.

## Introduction

Delta-opioid receptors (DOPrs) are expressed ubiquitously throughout the body and central nervous system and are, thus, positioned to play a role in a multitude of diseases. DOPr expression is often dynamic, with many reports of increased expression during exposure to some chronic states such as stress, inflammation, or neuropathy or ethanol consumption, and thus, agonists at DOPr become more potent and efficacious in mediating receptor-specific responses in these conditions. These combined features of DOPr pharmacology illustrate the potential benefit of designing tailored or biased DOPr ligands that could preferentially signal through certain specific signaling cascades (Zhang et al., 2006; Cahill et al*.,* 2007; Kabli and Cahill, 2007; van Rijn et al., 2012b; Conibear et al., 2020). According to some studies, DOPr activation does not result in many of the adverse effects associated with MOPr agonists, including addictive liability (Stevenson et al., 2005; Codd et al., 2009), respiratory depression (Takita et al., 1997; Codd et al., 2009), and constipation (Petrillo et al., 2003; Codd et al., 2009) and so could offer a promising alternative for the management of certain painful states such as cancer and peripheral neuropathy (Pradhan et al., 2010). A number of DOPr-selective agonists have been synthesized so far. The first generation of non-peptide compounds produced non-lethal convulsions in rodents and non-human primates, but subsequent generations of drugs were designed to avoid this problem. SNC80, a non-peptide agonist, produces convulsions at high doses, and therefore, it cannot be used clinically (Broom et al., 2002a; Broom et al., 2002b). The pro-convulsive effect of SNC80 was initially suggested to be mediated by DOPrs; however, no mechanistic link to the receptor has been established, and the new generation agonists such as PN6047 do not exhibit any proconvulsant activity. After prolonged exposure to PN6047 *in vitro* and in the rodent models, no analgesic tolerance or other opioid-mediated side effects were reported (Conibear et al., 2020).

Extracellular signal-regulated kinases, or ERKs, are widely expressed serine/threonine protein kinases, which function as mediators in intracellular signaling pathways. The ERK signaling pathway also plays a crucial role in multiple stages of tumorigenesis, including proliferation, migration, and invasion of cancer cells (Shaul & Seger, 2007; Kim and Choi, 2010). The MAPK pathways that have been characterized in mammals include the extracellular signal-regulated kinases (ERK kinases-1 and-2), Jun N-terminal kinases (JNK 1-3), and p38, each of which represents a separate signaling pathway (Pearson et al., 2001). The classical MAP kinase signaling pathway involves receptor tyrosine kinases such as growth factor receptors (e.g., epidermal, fibroblast, and tropomyosin receptor kinases), which activate a sequential signaling cascade resulting in ERK activation *via* phosphorylation of tyrosine residues (Cobb, 1999; Kholodenko, 2000). Activated ERK, in turn, phosphorylates a number of effectors, including transcription factors and other protein kinases. In addition to the classical pathway, ERK activity is also modulated by G protein-coupled receptors (GPCRs) *via* G protein and *β*-arrestin mediated pathways (May and Hill, 2008; Coffa et al., 2011), as well as integrins (Aplin et al., 2001; Lavoie et al., 2020), and noxious stimuli such as cytotoxic compounds and heat shock (Kyriakis & Avruch, 2001). GPCR-mediated ERK activation can be initiated by Ras, Rap, PKC, tyrosine kinases (e.g., c-Src), transactivation of receptor tyrosine kinases, or by *β*-arrestins (Wei et al., 2003; Ahn et al., 2004; Leroy et al., 200*7*)*.* MAPK modules contain 3-tier kinases that are sequentially activated by phosphorylation*.* Activated ERK1/2 are released from the three-kinase complex and phosphorylate approximately 600 cellular proteins (Yoon and Seger, 2006; Ünal et al., 2017), mediating a variety of cellular activities.

G protein-coupled receptors (GPCRs) are the most extensively researched therapeutic targets, owing to their importance in human pathophysiology and pharmacological versatility. GPCRs are thought to activate ERK in a variety of ways, including G-protein-dependent and G-protein-independent pathways. The role of ERK (activation) as a downstream effector of GPCR proteins, including the delta opioid receptor, is well documented. DOPr agonists induce receptor phosphorylation in a dose-dependent and agonist-selective manner, by which the different functional (downstream) effects of DOPr agonists are achieved (Mann et al., 2020).

Due to the important role of ERK signaling for a number of targets and effectors, it is increasingly seen as a primary functional measure of receptor activation in pharmacological research studies and drug discovery, as an alternative to more traditional assays such as cAMP accumulation (Leroy et al., 2007), and it is a target in its own right, particularly in pain and anti-cancer research (Roberts and Der, 2007; Kondo and Shibuta, 2020; Sugiura et al., 2021; Sun et al., 2021). ERKs are frequently hyperactivated in many types of cancer, which is usually induced by activating mutations of the K-Ras gene (Chatterjee et al., 2017). On the other hand, a number of studies have reported that DOPr and ERKs share the same downstream signaling pathways (Eisinger et al., 2002; Hong et al., 2009). According to a study conducted by Xu et al. (2010), DOPr activates ERK *via* G-protein or arrestin-dependent pathways.

The predominant experimental method for measuring ERK phosphorylation in cultured cells and other biological samples is Western blotting, in which target proteins are separated from prepared cell lysates by SDS-PAGE, transferred onto a polymer sheet, followed by detection with protein-specific antibodies (Mahmood and Yang, 2012). This technique has the advantage of being able to isolate and detect relatively small amounts of protein from whole cell samples and provides an estimate of the protein molecular weight. However, the technique involves numerous sensitive and time-consuming steps, which increase the potential for experimental variation. Sample preparation requires the destruction of cells by homogenization and/or lysis, and the solubilization of proteins with detergents,which results in protein samples that are far away from their native *in situ* environment. The technique is only semi-quantitative, and the number of samples that can be analyzed is constrained by the number of lanes available in commercially available phosphorylation immunoassay kits, which have the advantage of being simple and well-suited for routine screening (Ma et al., 2008) but are often prohibitively expensive and low-throughput in academic settings. Traditionally, screening assays were limited to a small number of targets, with many of them requiring purified proteins.

Over the last decade, high-throughput screening (HTS) laboratories have been more interested in cell-based high-content screens (HCS), which investigate biological events at a subcellular level. Currently, there are some plate-based techniques which could be used for HCS screening of agonists and antagonists, such as the AlphaScreen (Amplified Luminescent Proximity Homogeneous Assay)-ERK assay. It uses a bead proximity-based AlphaScreen technology which is robust, highly sensitive and involves no wash steps and is commonly used by the pharmaceutical industry to enable high-throughput screening of compounds for drug discovery (Eglen et al., 2007; Siehler, 2008). On the other hand, the Meso-Scale Discovery Assay, based on electrochemiluminesence, offers medium to high-throughput screening (Garbison et al., 2015). All these assays, in addition to LI-COR-In-Cell Western technology, could provide robust ERK estimations in pain drug discovery as well as measure ERK responses in tumors so that drug exposures can be associated with their effects on molecular biomarkers and efficacy. Another ERK activation assay, which is a BRET-based sensor of ERK modulation, called Rluc8-ERK substrate-Venus (REV) has been developed by Xu et al. (2013) to record BRET signals with an ERK-biosensor. A sandwich proximity-based phospho-ERK1/2 assay (384-well format) that utilizes homogenous time-resolved fluorescence technology has also shown great potential for cell-based HTS (Ayoub et al., 2014). However, delta opioids have not been tested on these screening platforms, so it will be interesting to develop a detailed optimization strategy for such compounds.

The LI-COR-In-Cell Western assay system was used in this work as a quantitative fluorescent immunocytochemical (ICC) assay, using an infrared (IR) scanner. The platform has been applied to a variety of macroscale analyses which allow for the detection of target proteins, including ERKs, in fixed cultured cells (Wong, 2004; Chen et al., 2005). In this instance, secondary antibodies are covalently linked to a near-infrared (IR) fluorophore, which can then be detected with an imaging scanner. The advantages of this technique are that ERK phosphorylation can be measured *in situ*, producing data of more biological relevance; assays can be performed in 96-or 384-well plates, allowing for high-content screening; and the values can be easily quantified as the total fluorescent signal from the cell population present in each well, allowing for more confident comparison of data points. Beyond ERK signaling, this fluorescent ICC technique can be used to measure post-translational modification and altered regulation of a wide array of proteins, with the only differing requirement being the target-specific primary antibodies.

We described a series of optimization steps for the fluorescence immunocytochemical phospho-ERK assay aiming to increase the sensitivity and reduce variance and cost, using the LI-COR-In-Cell Western system and hDOPr-mediated signaling in the recombinant CHO-K1 cell line, over-expressing the human delta-opioid receptor (hDOPr) as a model. In this study, we introduce a novel candidate compound, PN6047 (Conibear et al., 2020), in addition to some reference DOPr ligands (SNC80, ADL5859, and DADLE).

## Materials

The novel compound PN6047 was synthesized by our collaborator, PharmNovo AB (patent WO2016/099393, with US patent number 10,118,921 B2) (Conibear et al., 2020). The three standard selective agonists at DOPr: SNC80, ADL5859, and DADLE, and their selective antagonist, naltrindole, were obtained from Tocris Bioscience (Bristol, United Kingdom). Dulbecco’s Modified Eagle Medium: Nutrient Mixture F-12 (DMEM/F-12), fetal bovine serum L-glutamine, G 418 disulphate salt solution, PBS, bovine serum albumin, paraformaldehyde, Triton™ X-100, apyrase, adenosine 5′-triphosphate and reagents for I-CW were obtained from Sigma Aldrich (Poole, United Kingdom). Milk powder was obtained from the Co-operative (United Kingdom). Anti-phospho-ERK1/2 MAP kinase mouse (# 9106) and anti-ERK1/2 MAP kinase rabbit antibodies (# 9102) were obtained from New England BioLabs (Herts, United Kingdom). IRDye^®^ labeled goat anti-mouse (IRDye^®^ 800CW conjugate) and goat anti-rabbit (IRDye^®^ 680CW conjugate) secondary antibodies were obtained from LI-COR (Lincoln, NE). Pertussis toxin (PTx) was purchased from Calbiochem.

## Methods

### Cell culture and assay protocol

Levels of MAP kinase phosphorylation were determined using the In-Cell Western (I-CW) assay. The CHO-K1 cells, over-expressing the human delta-opioid receptor (hDOPr), were seeded into 96-well clear bottom plates at a seeding density of 25,000 cells per well (unless otherwise stated) in culture medium for 24–36 h at 37°C in a humidified 5% CO_2_/95% air atmosphere. The following day, the culture medium was replaced with serum-free media (SFM) and the cells re-incubated for 24 h at 37°C. The next day, serial dilution of DOPr ligands was performed in SFM and added to respective wells to a final volume of 200 µL per well. Assay plates were incubated for the stated period at 37°C during experimentation. After incubation, the assay medium was rapidly aspirated, and the cells were fixed with 4% paraformaldehyde (PFA) in PBS for 20 min at room temperature (100 µL per well). Each reagent was aspirated before the addition of the next reagent. Cells were then permeabilized with 0.1% (v/v) Triton^TM^ X-100 in PBS for 20 min at room temperature (100 µL per well). Non-specific antibody binding sites were blocked with 5% (w/v) milk powder in PBS for 20 min at room temperature with gentle rocking.

The two primary antibodies, for anti-total and anti-phosphorylated ERK1/2, were added to the blocking buffer (1:500) to form a primary antibody solution. Cells were incubated with primary antibody solution (100 µL per well) overnight at 4°C with gentle rocking. The next day, the primary antibody solution was aspirated, and the cells were washed three times with phosphate buffered saline (PBS) for 5 min at room temperature with gentle rocking. The appropriate LI-COR IRDye® secondary antibodies were diluted (1:500) in a blocking buffer, added to the cells (100 µL per well), and incubated in darkness for 1 h at 37°C with gentle shaking. For Phospho ERK1/2 secondary antibody, goat anti-mouse IRDye®800 (green) was used and with total ERK1/2 goat, we used anti-rabbit IRDye® 700 (red) which allowed simultaneous detection of both the proteins.

### Imaging

The secondary antibody solution was aspirated, and the cells were washed three times with wash buffer. The LI-COR Odyssey glass scanner surface was wiped with Millipore-Q water and the plate was scanned, using both 700 and 800 nm channels with a resolution of 169 μm, medium quality, an intensity of 3.0, and a focus offset of 4 mm. The data were analyzed by LI-COR Odyssey® application software (Image Studio).

### Data analysis

Blank wells consisting of cells and secondary antibody alone (without primary antibody solution) were used in this assay to estimate any background resulting from auto-fluorescence and/or any fluorescence from non-specific binding of the secondary antibody. Therefore, the responses generated from blank wells were subtracted from the secondary antibody responses to give a measure of non-specific binding of the secondary antibody. Using the Odyssey automated system, each band was converted to arbitrary numbers by placing a circle around each well on a scale of 0–1 (0 being the lowest, and 1 being the highest intensity, detected on IR). The numbers generated from the 700 nm wavelength represented the total expression of ERK within all the cells of the well. The arbitrary numbers generated from the 800 nm wavelength represented the phosphorylated ERK in the well. To eliminate the well-to-well cell seeding variation, the signal was converted to the amount of phosphorylation per single arbitrary unit of total ERK (normalized ratio = phospho ERK/total ERK, i.e., 800/700 nm). Curve-fitting (nonlinear regression) and statistical analyses were performed using GraphPad Prism 8.01 (San Diego, CA).

Each data point represents the mean ± SEM of at least 3-5 experiments performed in triplicate. EC_50_ is the molar concentration of agonist required to generate a response of 50% of E_max_, whereas E_max_ is the maximum response produced by each agonist relative to the maximum response of the designated standard agonist SNC80 (= 100%).

## Results

In this study, we have reported validation of the I-CW-Phospho-ERK assay to investigate the intracellular ERK1/2 signaling pathway, compatible with HTS in a 96-well plate format. A number of experimental conditions, including edge effect, plate type, incubation procedure, cell seeding densities, effect of serum starvation, and mechanism of DOPr action, were investigated. We also reproduced the assay protocol and optimization steps in order to generate concentration-response curves for the DOPr agonists, as well as determine their potency and efficacy.

### Reduction of edge effect

**FIGURE 1:**
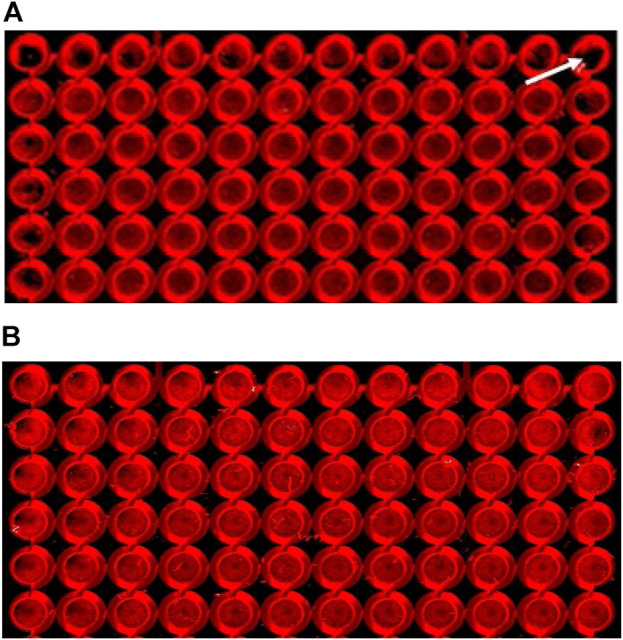
Cells pre-incubated at room temperature for 30 min displayed a reduced edge-effect. Cells were assayed as described in the Methods section, using SNC80 (1 µM) to activate DOPr. Images of 96-well plates seeded with 25,000 cells per well were placed either **(A)** immediately into a 37°C incubator (the white arrow indicates a peripheral well with a marked edge effect), or **(B)** pre-incubated for 30 min at room temperature (22 ± 1°C), prior to incubation at 37°C. Images show cells cultured for 24 h, fixed, permeabilized, blocked, and labeled with primary (ERK1/2) and secondary (IRDye^®^) antibodies. The images were scanned using the LI-COR Odyssey^®^ system (Image Studio). Effect of DOPr agonists on ERK activation time-course.

Preliminary I-CW experiments clearly demonstrated an uneven distribution pattern of cells in the perimeter wells of the 96-well microplates when scanned. Differential evaporation rates and thermal changes in the plate lead to edge effects, thus causing heterogeneity or variability in results. Upon microscopic inspection, cells in the outer wells of the plate appeared to be clumped toward the outer edges of the wells (Figure 1A). Pre-incubation of the plates at room temperature (RT) for 30 min immediately after seeding and prior to moving the plates to the incubator markedly decreased the visible edge effect (Figure 1B). A nearly similar effect was achieved when 200 µL/well of blank/DMEM/F12 media was added to the peripheral wells during the cell seeding step, thus helping to control the rate of evaporation from the vulnerable wells (not shown). Another method to reduce the edge effect was tested by using a specialized “edge 96-well plate” (from Thermo Scientific Nunc) with a surrounding moat which, when filled with blank media, acts as an evaporation buffer during longer incubations. This modification gave very similar results (data not shown) to those of the 30-minute pre-incubation at room temperature approach. Since 30 min of pre-incubation at room temperature (RT) in our experiments led to a fairly uniform distribution of cells across the peripheral wells of our cell line, this method was used for all of the experiments in this study.

**FIGURE 2:**
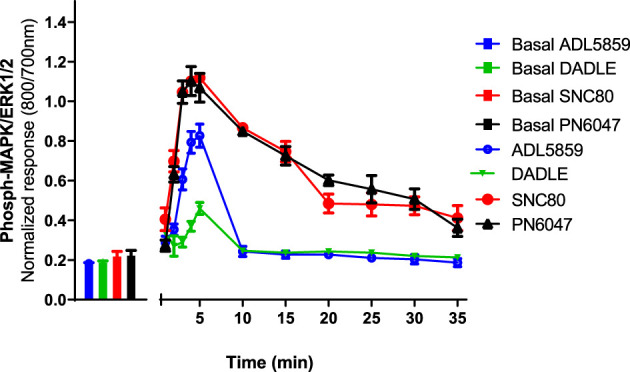
Time-course of the effect of DOPr agonists (SNC80, ADL5859, DADLE, and PN6047) on ERK phosphorylation in CHO-K1-hDOPr. Cells were cultured in the DMEM/F12 medium for 24 h, followed by serum-starvation for 24 h. Cells were stimulated with DOPr agonists (10 µM) for the indicated times, followed by rapid fixation with PFA (4% w/v) solution. Detection of total and phospho-ERK levels was carried out as stated in the Methods Section. Individual ERK phosphorylation values were normalized to the corresponding total-ERK values. The bars show basal ERK responses from unstimulated cells. The data shown are mean ± SEM values from 5 independent experiments performed in triplicate.

CHO-K1-hDOR cells were treated with 10 µM concentrations of SNC80, ADL5859, DADLE, and PN6047 at varying time points (1–35 min), and the phospho-ERK responses were measured. This temporal characterization was important as subsequent experiments were carried out on the basis of these results. Optimal agonist stimulation time points also provided a means to identify any multiphasic responses. ERK phosphorylation was detectable as early as 1 min after agonist addition, with a distinct peak at 3–5 min (Figure 2). SNC80, ADL5859, and DADLE responses declined sharply after 5 min when compared with the baseline. The response afterwards steadily declined for up to 35 min. In further experiments, a 5-minute time period was chosen for ERK activation.

### Cell density quantification

**FIGURE 3:**
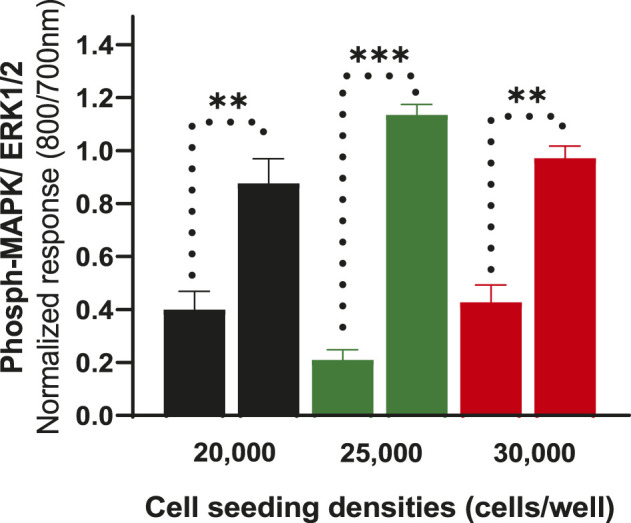
Effect of cell seeding densities (20,000, 25,000 and 30,000 cells per well) expressed as normalized data showing values for basal and their corresponding SNC80 stimulated ERK phosphorylation. The data shown are the means of triplicate measures ± SEM. Statistical significance was determined using a paired two-tailed t-test; **p < 0.01, ***p < 0.001.

To further determine whether cell density affects ERK phosphorylation of CHO-K1-hDOPr cells, cell seeding densities were optimized to evaluate assay robustness and performance. The cells were plated at different densities (20,000, 25,000, and 30,000 cells per well) into 96-well microtiter plates for 24 h. Cells were serum-starved for another 24 h and then stimulated with 10 μM SNC80, which produced an increase in ERK phosphorylation at all cell seeding densities tested, with no significant differences between conditions. The wells were not fully confluent when seeded with 20,000 cells per well. The cells appeared over-confluent when inspected after 24 h at seeding densities of 30,000/well and showed higher basal values. The cells, on the other hand, produced a much cleaner image, as they were uniformly distributed across all the wells when plated at 25,000 cells per well (image not shown). Also, the basal levels of ERK phosphorylation for 25,000 cells seeded per well were markedly lower than for the other seeding densities, with a significantly higher ERK activation (one-way ANOVA) (Figure 3). This cell number was chosen for further experimentation.

### Black versus clear-walled assay plates

**FIGURE 4:**
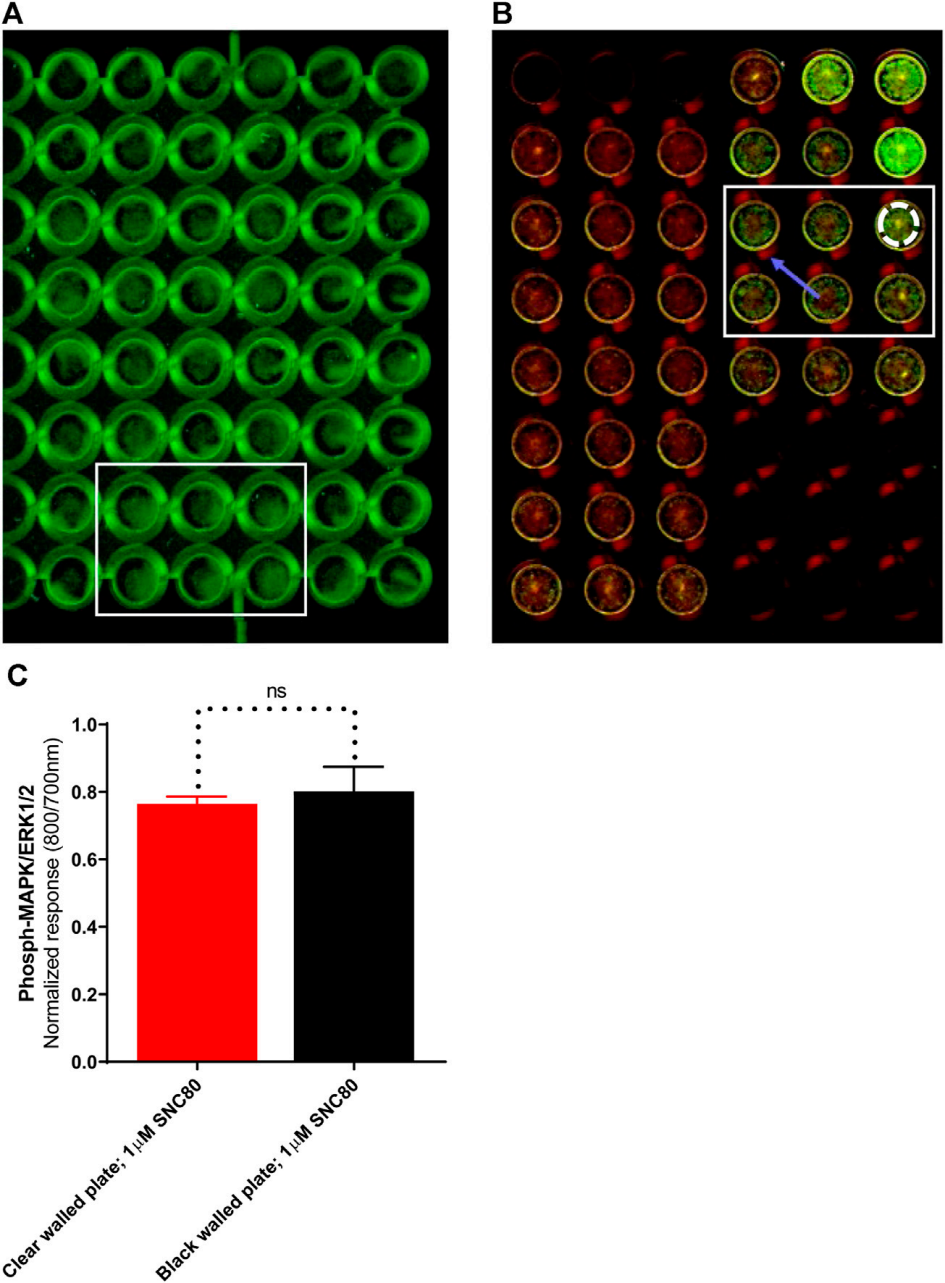
Images of randomly selected wells from **(A)**black-walled or **(B)** clear-walled 96-well plates seeded with 25,000 cells/well. The blue arrow indicates the presence of scattered light near-infrared fluorescence in the dead zone between the wells. **(C)** Phospho-ERK response in SNC80 treated cells in clear and black-walled assay plates, stimulated with 1 µM; 5 min). ns *p* > 0.05 (unpaired two-tailed t-test). Differential effects of DOPr agonists in the presence or absence of serum.

LI-COR standard protocols recommend using black-walled assay plates to prevent potential light leakage between neighboring wells. Scanning of the plates in clear-welled assay plates revealed light scattering observable in the dead zone between wells (Figure 4B; blue arrow). However, because quantification includes measuring intensity in a discrete area of each well using a template superimposed onto the scan frame (Figure 4B; white dashed circle), light bleed between adjacent wells is unlikely to be a significant problem. As a result, the impact of plate type (black versus clear-walled; plate 4A vs. 4B, respectively) was investigated. There was no significant difference (*p* > 0.05, unpaired t-test) between the two plate types in the ERK phosphorylation response to stimulation with the selective delta-opioid agonist, SNC80 (see randomly selected wells/rectangular area stimulated with 1 µM; 5 min) between the two plate types (Figure 4C).

**FIGURE 5:**
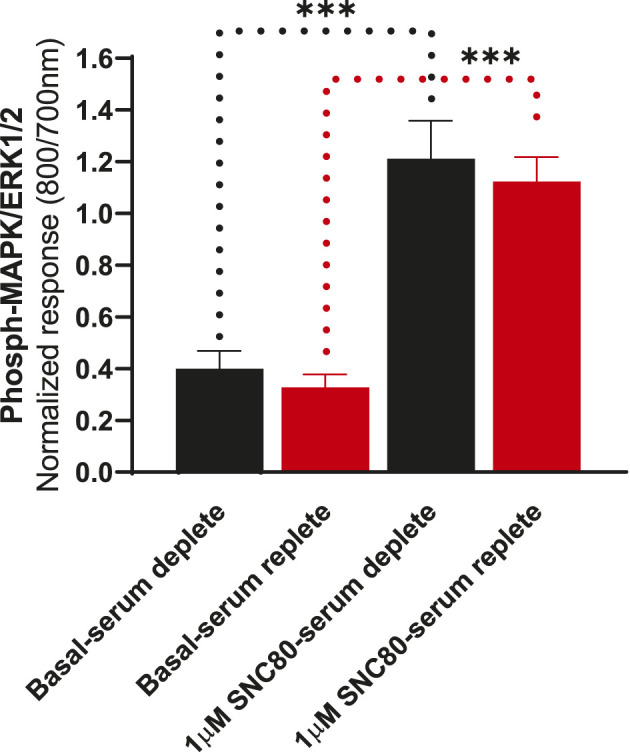
Differential effects of SNC80 (1 µM) on ERK activation in serum-replete (10% FBS) versus serum-depleted (no FBS) conditions in CHO-K1-hDOPr cells. The signal is expressed as a normalized ratio (800/700 nm) of phosphorylated to total ERK1/2. The data shown are the mean ± SEM values from three independent experiments performed in triplicate. Statistical significance was determined using one-way ANOVA; ***p < 0.001. Extracellular ATP as a contributor to increased basal ERK signaling.

To determine whether the presence of serum alters the effects of DOPr agonists on MAPK activation, we compared the activation of ERK following overnight incubation in serum-replete (10% FBS) versus serum-depleted (no FBS) conditions (Figure 5). There were no significant differences in either basal or SNC80-stimulated ERK phosphorylation between serum-containing and serum-depleted conditions in CHO-K1-hDOPr cells (one-way ANOVA).

**FIGURE 6:**
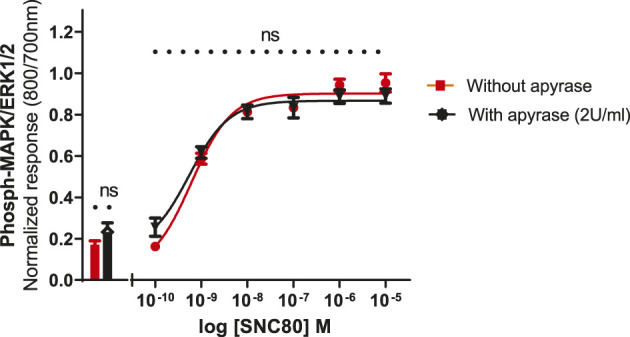
Effect of apyrase on phospho-ERK response (basal and SN80-stimulated ERK phosphorylation) in the absence or presence of 2 units/ml of apyrase incubated for 30 min. Individual ERK phosphorylation values were normalized to the corresponding total-ERK values. The data shown are the mean ± SEM values from four independent experiments performed in triplicate. The bars show basal ERK responses from unstimulated cells. No statistical difference was observed between the two responses (with and/or without apyrase) (ns *p* > 0.05; unpaired two-tailed t test).

To see whether basal phosphorylation levels could be reduced by apyrase (an ATPase) or if it might contribute to an increase in the size of the signal, experiments were performed in the presence or absence of apyrase. Based upon previous studies in other cell lines, it has been suggested that the ERK1/2 basal levels could be significantly reduced by the inclusion of apyrase, suggesting a possible involvement of extracellular nucleotide signaling, i.e., endogenous ATP release from the cells (Burnstock et al., 2010). Therefore, the cells were pre-incubated with apyrase (2 units per ml) for 1 h at 37°C and assayed as described in the Methods section. Apyrase, however, did not significantly reduce the basal levels of ERK phosphorylation (unpaired two-tailed t-test), implying that basal phosphorylation levels were not regulated by ambient concentrations of extracellular ATP in this cell line. Similarly, apyrase did not have a significant effect on SNC80-stimulated ERK phosphorylation (Figure 6).

### ERK activation, concentration–response curves

**FIGURE 7:**
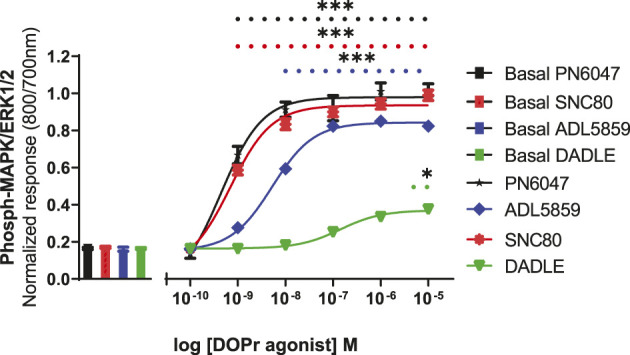
Effect of DOPr agonists: SNC80, ADL8859, DADLE, and PN6047, on ERK phosphorylation in CHO-K1-hDOPr cells. Cells were cultured in growth medium for 24 h, followed by serum starvation for 24 h. Cells were stimulated with various concentrations of DOPr agonists for 5 min, followed by rapid fixation with PFA solution (4% w/v). Detection of total and phospho-ERK levels was carried out as stated in the Methods section. The bars show basal ERK responses from unstimulated cells. The data shown represent the mean ± SEM of four experimental repeats, each run-in triplicate. Statistical significance was determined using an unpaired two-tailed t-test; *p < 0.05, ***p < 0.001.

A stimulation period of 5 min was chosen for DOPr-agonist concentration-response experiments, representing the peak of the first phase response. All agonists produced a concentration-dependent increase in phospho-ERK levels (Figure 7). We also calculated the potencies and efficacies of the DOPr ligands. The rank order of potencies (logEC50 values) of the agonists acting on CHO-K1-hDOR cells from highest to lowest was SNC80 > PN6047 > ADL5859 > DADLE. With regard to the three standard agonists, DADLE (a peptide) was clearly much less effective than the non-peptides, with an E_max_ of less than 40% of the other two agonists (one-way ANOVA) (Table 1).

**TABLE 1:** Estimates of EC50, logEC50, and Emax obtained from fitting the three-parameter sigmoid curve.

DOPr agonist	EC_50_ (nM) ± SEM	LogEC_50_ ± SEM	E_max_ ± SEM (% SNC80)
SNC80	1.6 ± 0.3	−9.0 ± 0.17	100 ± 3
ADL5859	8.0* **±** 1.5	−8.0 ± 0.01	100 ± 1
DADLE	86* **±** 4.9	−7.0 ± 0.01	36* ± 1
PN6047	1.4 **±** 0.2	−8.7 ± 0.02	102 ± 1

Potency (EC_50_, logEC_50_) and efficacy (E_max_) values for DOPr agonist activation of ERK1/2 in CHO-K1-hDOR cells. Shown are the mean values ± SEM from four separate experiments, performed as detailed under the Methods section, with the maximum response induced by SNC80 defined as 100%. *p < 0.05 shows significantly less potency or efficacy than SNC80; one-way ANOVA, followed by Dunnett’s multiple comparisons test.

### Mechanism of hDOPr-induced ERK activation

**FIGURE 8:**
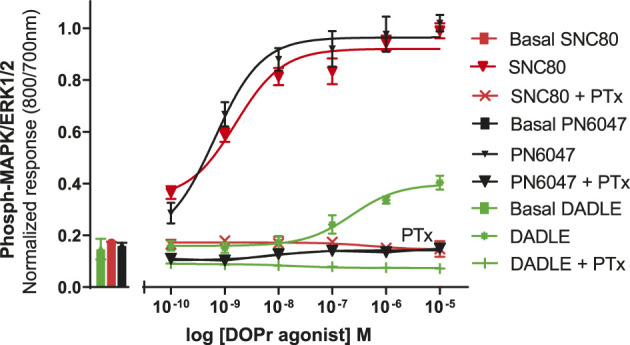
Effects of DOPr agonists on ERK phosphorylation in PTx-treated CHO-K1-hDOPr cells. 25,000 cells per well were cultured for 24 h, followed by serum starvation for 24 h, in medium containing 100 nM PTx. Cells were stimulated with different concentrations of SNC80, DADLE, and PN6047 for 5 min, followed by rapid fixation with PFA (4% w/v) solution. Detection of total and phospho-ERK levels was carried out as stated in the Methods section. The bars show basal ERK responses from unstimulated cells. The data shown are the means ± SEM values from 3 independent experiments performed in triplicate.

**FIGURE 9:**
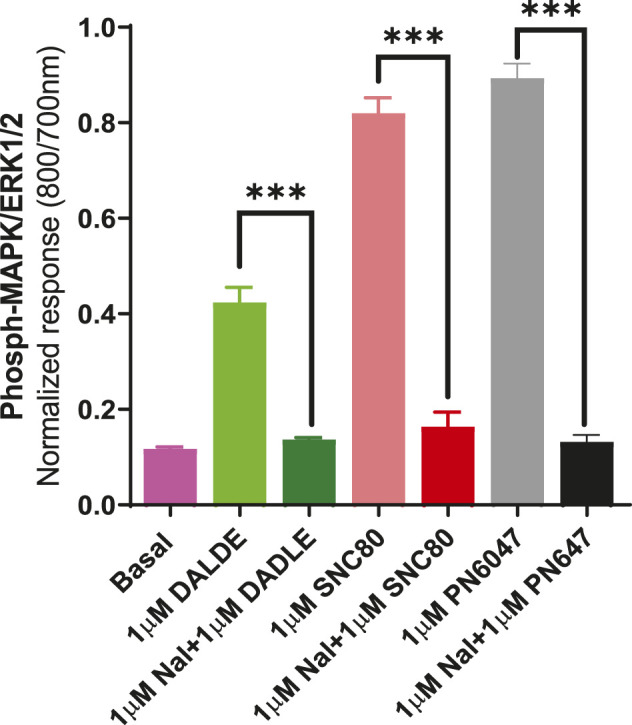
Effect of the selective DOPr antagonist, naltrindole, on agonists’ action. 1 µM concentrations of each of the DOPr ligands (DADLE, SNC80, and PN6047) were co-incubated with an equal concentration of naltrindole in CHO-K-1- hDOPr cells. The data shown are the means ± SEM values from 3 independent experiments performed in triplicate. Statistical significance was determined using one-way ANOVA; ***p < 0.001.

a) To investigate the performance of the assay in determining the role of Gi/o proteins in the DOPr-mediated phospho-ERK responses, the experiments were carried out in CHO-K1-hDOPr cells with overnight incubation.A 100 ng/ml pertussis toxin (PTx), a Gi/o protein blocker, was added, followed by exposure to DOPr agonists. The result showed that PTx abolished the DOPr-mediated ERK phosphorylation responses (Figure 8). The loss of ERK activation suggested that the enhanced MAPK activation was mediated by a PTx-sensitive Gi/o protein in the system (Mangmool and Kurose, 2011).
b) To further establish the role of DOPr in the agonist responses, the experiments were carried out in the absence and presence of the DOPr-selective competitive antagonist naltrindole (1 µM). Naltrindole blocked the stimulatory effects of the DOPr agonists: SNC80, DADLE, and PN6047, as shown in Figure 9.

## Discussion

ERK is an important, ubiquitous signaling mediator, with ERK phosphorylation an increasingly used measure of receptor function both *in vitro* and *in vivo*. Plate-based immunocytochemical assays provide an excellent method of quantifying ERK phosphorylation in a cheap and relatively high-throughput manner. In this article, we have used the LI-COR In-Cell Western system to measure phospho-ERK responses. However, alternative detection techniques, such as secondary antibodies tagged with fluorescein and/or rhodamine-based fluorophores on plate readers, are equally effective. The optimizations presented here are, to the best extent possible, independent of the detection method employed and aimed to increase assay sensitivity while minimizing variability and cost.

Our group has identified the novel small-molecule PN6047, which has demonstrated oral bioavailability. The discovery of PN6047 was based on G protein-bias at DOPr and displaying partial agonism in the arrestin recruitment and internalization assays. In preclinical models of chronic pain, PN6047 demonstrated potent antihyperalgesic activity. Moreover, PN6047 does not appear to cause analgesic tolerance or have proconvulsive activity (Conibear et al., 2020). In addition to using other detection methods such as cAMP, arrestin recruitment, and internalization assays, we decided to test our ligands in the I-CW/ERK assay to confirm whether they could be used as an efficient model in the drug discovery process. Once the I-CW assay was assessed as a suitable choice to transfer into HTS, the next step was to optimize and validate the assay with all the available standard DOPr ligands. Using this data, we modeled all the necessary assay cut-offs. Multi-channel dispensers and aspirators were used to make this process faster. The throughput of the assay was determined, and once the repeatability and reliability of the assay were confirmed, we tested our novel compound, PN6047. The paradigm was also tested with a number of other potential DOPr ligands that hold promising preclinical efficacy (data not shown).

Prior to conducting the I-CW assay, the standard ligands SNC80 and DADLE were subjected to Western blot (WB) analysis (Conibear et al., 2020), in which the activation (phosphorylation) of ERK1/2 proteins was confirmed. However, we suggested and preferred the I-CW assay over conventional WB to avoid cumbersome operational steps in WB, keeping in view that generation of concentration-response curves for large libraries of agonists was more practicable with the I-CW assay. The I-CW/ERK assay paradigms have been used successfully by our group to identify a number of hit and candidate molecules, including the novel DOPr agonist, PN6047, which is a G protein-biased and selective DOPr agonist with analgesic efficacy in models of chronic pain. In cell-based assays, PN6047 fully engages G-protein signaling but is a partial agonist in both the arrestin recruitment and internalization assays (Asghar, 2017; Conibear et al., 2020).

In recent years, the engineering of biosensors tailored to different signaling pathways has been donein an effort to explore the dynamic biological processes. For example, the development of biosensors based on fluorescent proteins has enabled researchers to measure ERK activity in living cells. Genetically encoded resonance energy transfer technologies (Fluorescent, FRET, or Bioluminescent, BRET)-based biosensors have proven to be efficient in locating and quantifying enzyme activities, such as subtle variations in ERK activities. The spatiotemporal regulation of kinases is of particular importance since it is essential for a thorough understanding of cell fate. This is particularly the case for ERK, whose activity serves as a crucial component in signal transduction pathways and can guide the cell towards a variety of cellular behaviors (Xu et al., 2013; Hirata and Kiyokawa, 2019).

An ideal assay for GPCR ligand screening should be simple, non-radioactive, robust, and easily adaptable to a microtiter plate format. I-CW is an immunofluorescence-based technique in which the target protein is probed with epitope-specific antibodies in fixed cultured cells in 96-well plates (Daigle et al., 2008). As compared to conventional Western blotting, I-CW is a pharmacologically more conducive and suitable assay for screening libraries of compounds. It is important to highlight that no previous studies have used I-CW assays to measure ERK responses to DOPr agonists; hence, it was necessary to evaluate its feasibility in our cell line.

Preliminary experiments revealed a clearly noticeable edge effect in the assay plates’ peripheral wells. Evaporation can occur in all the wells in long-term cultures but is more frequent in corner wells as they are more exposed to the surroundings, and this effect can frequently be seen in 1–3 days of culture. This may also cause culture problems in some sensitive cell types since the medium components, especially the salts, may reach concentrations that are harmful to the cells. It is possible to reduce the evaporation by controlling humidity in the CO_2_ incubators and limiting the number of outside inspections, as opening the incubator door too frequently is a contributing factor to high rates of evaporation. Moreover, we clearly found fewer volumes of medium in the peripheral wells of the plate, so one intervention could be to add larger volumes of medium in perimeter wells to compensate for the lost water. In the present study, however, we compared the edge effect using pre-incubation of the plates at room temperature (in the laminar hood) for 30 min immediately after seeding and prior to moving to the incubator and found that this markedly decreased the visible edge effect (Figure 1). This method was reported by Lundholt et al. (2003) to control edge effects on microplates. A similar effect was achieved when cells were grown in the inner wells and blank DMEM/F12 media was added to the peripheral wells as a measure to allow water loss from the perimeter wells, thus controlling the rate of evaporation from the inner wells. However, this results in the loss of a number of data points on each plate, so we did not proceed with this approach. We also tested the possible advantages of using a specialised “edge 96-well plate from Thermo Scientific Nunc”, with a surrounding moat which, when filled with blank media, acts as an evaporation buffer during longer incubations. This intervention gave comparable results to that of a 30-minute pre-incubation at room temperature. However, the results obtained from 30 min of pre-incubation at RT indicated a fairly uniform distribution of the cells across the plate at a lower cost. Therefore, all the experiments in this study were performed using this intervention. However, there is still room for optimizing the pre-incubation time for other cell lines.

Multiphasic ERK signaling *via* GPCRs is a commonly observed phenomenon, which can be mediated by G proteins, *β*-arrestins, and other scaffolding proteins (Kolch, 2005), as well as *via* transactivation of receptor tyrosine kinases (Wetzker and Bohmer, 2003). The results showed that SNC80 produced a significantly high phospho-ERK response in CHO-K1-hDOPr cells between 3–5 min of agonist stimulation. MOPr and KOPr stimulation have been demonstrated to initiate ERK1/2 phosphorylation in astrocyte cultures and transfected cell lines. The DOPr selective agonist, DPDPE, has also been shown to activate ERK1/2 through G*βγ* and Ras signaling cascades. The kinetics of ERK1/2 phosphorylation by MOPr and KOPr systems vary, yet both receptors can activate ERK1/2 within 5–10 min (Belcheva et al., 1998; Al-Hasani and Bruchas, 2011). Our agonists’ responses showed a distinct peak between 3–5 min, following which they started to fall significantly, presumably due to phosphatase activation, and continued to decline gradually for the remaining observation window of up to 35 min (Figure 2). Due to the highest ERK response, we decided to stimulate our cells for 5 min in all the experiments. Since sustained ERK activation could be important in determining phasic agonist responses (e.g., receptor desensitization), it would be interesting to determine the time-courses over an extended period of time.

Optimization of the cell seeding densities showed that 25,000 cells per well gave the lowest basal phospho-ERK level and a much cleaner agonist ERK1/2 signal (Figure 3). Interestingly, the cells reached 100% confluency in 24 h with this cell seeding number, whereas the other cell numbers were either under-or over-confluent. Notably, the E_max_ of the agonist (SNC80) seemed to change/vary depending on the number of cells that were seeded (data not shown), emphasizing the need to maintain as nearly identical experimental conditions as possible when comparing the properties of different agonists in signaling assays.

Next, the standard protocol for In-Cell Western assays recommends the use of clear-bottom, black-walled plates to minimize crosstalk between the neighboring wells. However, these plates are typically more expensive than all-clear plates, which may prove to be prohibitive. The use of clear 96-well plates displayed a scattering of fluorescence signals into dead space between wells. However, as far as we can demonstrate, this failed to influence the values generated on the LI-COR scanner, and no statistically significant change in signal was observed (Figure 4). Moreover, since the results of each well are quantified by the use of a discrete template superimposed on the scanned image, the scattered light can be easily excluded.

All cell lines, including CHOs, endogenously express an array of receptors that also signal *via* MAP kinases, which is why cells were tested in both serum-free and serum-replete conditions, but the basal and agonist-stimulated phospho-ERK levels in both the conditions were not statistically different (Figure 5). It is speculated that the lack of a change in basal levels of ERK1/2 phosphorylation after longer periods of serum replete conditions is due to phosphatases degrading any excess ERK1/2 (Martín et al., 2005), thereby returning basal levels to similar levels as demonstrated in serum-free conditions. Although it is believed that serum restriction (growth factor deprivation) will reduce the basal activity of the cells, results from various experimental studies do not support this notion (Pirkmajer and Chibalin, 2011). Nevertheless, it has become a traditional laboratory practice to achieve more reproducible experimental conditions by subjecting serum restrictions. Furthermore, serum starvation supposedly reduces basal cellular activity and makes the population of proliferating cells more homogeneous, but serum deprivation has also been referred to as an environmental stressor and an apoptotic trigger (Bousette et al., 2010; Levin et al., 2010). Serum deprivation is frequently employed as an apoptotic strategy in HEK293 cells. We also speculated that serum-free conditions may be more pronounced and relevant in some cell lines (cell type-dependent responses). A unifying molecular explanation is elusive due to the fact that several signaling pathways have exhibited markedly opposing changes in different cell lines. Furthermore, it is proposed that the duration of serum deprivation be experimentally determined at several time points for each cell line in order to explore the implications of a particular assay in greater depth. Although serum starvation has proved to be a useful tool in cell signaling research, the physiological extrapolations of data obtained from serum starved cells must be subjected to constant scrutiny. In line with the general consensus, in this study, we decided to serum starve the cells prior to stimulation with DOPr agonists in further experiments to exclude any “endogenous” ERK responses released from the cells that could cause interference with the ligand responses.

Interestingly, adding vehicle to test wells without agonist (as a negative control) elicited a minor phospho-ERK response in the CHO-hDOPr cells, albeit this was adjusted for by expressing the data as additions over baseline. The possible cause of this vehicle-mediated response was not, however, investigated. Many cells, including neuronal cells, release ATP in the culture after even very mild stimulation (such as tilting of plates, vehicle and/or drug addition to wells) and thus are involved in the propagation of calcium waves and activation of numerous signaling pathways. All this could lead to changes in the cellular behavior and discrepancies in the results’ outcomes (Ostrom et al., 2000; Insel et al., 2001; Coco et al., 2003; Darby et al., 2003; Schwiebert and Zsembery, 2003; Gomes et al., 2005; Henriksen et al., 2006), so the addition of the ADP/ATP hydrolase enzyme apyrase was examined since this has been previously shown to reduce basal ERK phosphorylation in some cell lines. However, it was observed that apyrase had no significant effect on either basal or DOPr agonist stimulated ERK activation (Figure 6).

We also investigated the inhibition of ERK1/2 activation with the Gi/o protein blocker, pertussis toxin (PTx) (Figure 8). PTx inhibits Gi/o proteins (Mangmool and Kurose, 2011), suggesting that the loss of ERK activation was mediated by a PTx-sensitive Gi/o protein. The most convincing evidence that opioid receptors predominantly belong to the Gαi-coupled GPCRs comes from their sensitivity to PTx, which inactivates (by ADP-ribosylating) the Gαi subunit of the signaling complex, thus removing the inhibitory component of the pathway and causing blunting of agonist signals (Jiang and Bajpayee, 2009). The DOPr-mediated responses were also confirmed by using naltrindole, a highly selective and potent non-peptide delta opioid receptor antagonist, which abolished the ERK phosphorylation in our CHO-K1-hDOPr cell line (Figure 9).

All agonist-mediated phospho-ERK responses at 5 min were clearly shown to be concentration-dependent (Figure 7). A clear pattern of ERK mediated partial agonism was observed when comparing the E_max_ value of DADLE, which was significantly less efficacious than SNC80 and PN6047. The peptide DADLE’s lessened ERK activation may be due to the fact that it is a partial agonist at DOPr. Our Western blot, cAMP, and *β*-arrestin assays also exhibited the same partial responses (data not shown). Furthermore, these findings agree with our previously published data in which DADLE showed partial effects in the Western blot assays performed on HEK293 cells, transiently transfected with h-DOPr (Conibear et al., 2020). E_max_ values depend not only on ligand properties but also on system determinants such as total receptor number and maximal response allowed by the system. When compared with the E_max_ of SNC80, the novel compound PN6047 and the other standard ADL5859 were full agonists, and the rank order of agonists’ potency was SNC80 = PN6047 > ADL5859 > DADLE. The In-Cell Western assay proved to be an effective tool for measuring DOPr-mediated ERK1/2 signaling. All of the agonists in the set examined enhanced ERK phosphorylation, with PN6047 showing comparable ERK activation to that of SNC80, and this is in agreement with our previous findings on HEK-DOPr stable cell line using Western blotting (Conibear et al., 2020). All the ligands displayed a range of efficacies as well as widely different potencies. Given that a high-expression DOPr cell line was used in the assay, the low intrinsic efficacy of DADLE in ERK assays cannot be attributed to the low receptor reserve of the system. However, care must be exercised over such an interpretation as the different assay conditions or cell line/environment could have a major influence on the calculated potency and efficacy values.

### Future directions

As a future strategy, we recommend comparing the sensitivity and throughput of the I-CW/ERK assay against other high-content ERK signaling platforms, such as the ALPHAscreen (Eglen et al., 2007; Siehler, 2008), and/or the cell-based Phospho-ERK1/2 assay, using the homogeneous time-resolved fluorescence technology, reported by Ayoub et al. (2014). The same could possibly be studied in living cells/neurons by employing BRET-based ERK modulation assays (Xu et al., 2013). This all will, of course, require extensive optimization of conditions for the delta-opioids’ screening.

## Conclusion

We have described a series of optimization steps and other considerations to a standard protocol for fluorescence immunocytochemical assays measuring ERK phosphorylation using the In-Cell Western system. The optimized method presented here has allowed a relatively comprehensive pharmacological characterization of some delta-opioid receptor agonists, and we proposed that this approach could be applied to the other receptor systems. The I-CW/ERK assay is powerful because it allows researchers to investigate a wide range of agonist-directed and biased signaling, with ERK1/2 activation being one of the most crucial signaling pathways having substantial physiological and pathophysiological implications. The I-CW assay is a powerful substitute for western blotting. This assay system does not require time-consuming, error-prone steps like cell lysis, gel electrophoresis, or membrane transfer. In these experiments, DOPr overexpressing cells were used, which are very different, in all probability, from the real target neurons in the CNS and peripheral nervous systems. However, they are, at least, a homogenous source of recombinant human DOPrs in the high-throughput assays when investigating signaling profiles of libraries of novel compounds in lead optimization studies. The present study also provides a framework to systematically compare libraries of GPCR ligands, thereby allowing the classification of compounds into distinct clusters, which could have important implications in drug discovery and therapeutic development directed against chronic pain and certain neurological conditions.

ERK is a key cellular effector activated by GPCR agonists. ERK activation can be mediated by either the G protein or the *β*-arrestin signaling pathways. The subcellular location of activated ERK1/2 may differ depending on the activation pathway. G-protein-dependent ERK activation results in the translocation of active ERK to the nucleus, whereas *β*-arrestin-dependent ERK activation remains primarily in the cytoplasm. Many ERK1/2 substrates are located in the nucleus, including nuclear transcription factors involved in gene transcription, cell proliferation, and differentiation. ERK1/2 substrates are also found in the cytosol and other cellular organelles, where they may play roles in translation, mitosis, apoptosis, and signaling crosstalk. As a result, knowing the subcellular sites of activated ERK1/2 mediated by GPCR ligands would be critical in linking signaling pathways to cellular functions. While GPCR-stimulated selective ERK pathway activation has been explored in multiple receptor systems, so therapeutic exploitation of these diverse signaling cascades has yet to be significantly pursued. Because ERK activation is a critical signaling system that is linked to a variety of physiological tasks, targeting the ERK pathway may offer new avenues for GPCR drug discovery (Eishingdrelo and Kongsamut, 2013).

The rapid advances in the field of drug discovery and biased nature of the compounds have encouraged the demand for designing high-throughput screens (HTS) to characterize and optimize lead compounds. It is crucial to test the efficacies and potencies of novel ligands in various assays because it is known that DOPr agonists display biased signaling towards certain pathways. If a functional assay capturing only one signaling pathway is selected for screening, potentially valuable compounds may be missed if that compound exhibits biased signaling. Therefore, development of sophisticated high-content and robust cell-based functional assays can provide more accurate and comprehensive *in vitro* characterization of compounds targeting GPCRs.

## Data Availability

The raw data supporting the conclusion of this article will be made available by the authors, without undue reservation.

## Abbreviations

ADL5859, N,N-diethyl-4-(5-hydroxyspiro[2H-1-benzopyran-2,4′-piperidin]-4-yl)-benzamide; CHO-K1-hDOPr, Chinese hamster ovary cells over-expressing the human delta-opioid receptor; DMEM/F-12, Dulbecco’s Modified Eagle Medium: Nutrient Mixture F-12; DOPr, delta-opioid receptor; DADLE, [D-Ala2, D-Leu5]-enkephalin; GPCR, G protein-coupled receptor; HTS, high-throughput screening; I-CW, In-Cell Western; PN6047, 3-[[4-(dimethylcarbamoyl)phenyl]-[1-(thiazol-5-ylmethyl)-4-piperidylidene]methyl]benzamide; SNC80, 4-(alpha-(4-allyl-2,5-dimethyl-1-piperazinyl)-3-methoxybenzyl)-N,N-diethylbenzamide.
